# Fermented foods: a perspective on their role in delivering biotics

**DOI:** 10.3389/fmicb.2023.1196239

**Published:** 2023-05-12

**Authors:** Gabriel Vinderola, Paul D. Cotter, Miguel Freitas, Miguel Gueimonde, Hannah D. Holscher, Patricia Ruas-Madiedo, Seppo Salminen, Kelly S. Swanson, Mary Ellen Sanders, Christopher J. Cifelli

**Affiliations:** ^1^Instituto de Lactología Industrial (CONICET-UNL), Faculty of Chemical Engineering, National University of Litoral, Santa Fe, Argentina; ^2^Teagasc Food Research Centre, Moorepark and APC Microbiome Ireland, Cork, Ireland; ^3^Health and Scientific Affairs, Danone North America, White Plains, NY, United States; ^4^Department of Microbiology and Biochemistry of Dairy Products, Instituto de Productos Lácteos de Asturias—Consejo Superior de Investigaciones Científicas (IPLA-CSIC), Villaviciosa, Asturias, Spain; ^5^Department of Food Science and Human Nutrition, Division of Nutritional Sciences, 260 Edward R. Madigan Laboratory, University of Illinois, Urbana, IL, United States; ^6^Functional Foods Forum, Faculty of Medicine, University of Turku, Turku, Finland; ^7^Department of Animal Sciences, University of Illinois at Urbana-Champaign, Urbana, IL, United States; ^8^International Scientific Association for Probiotics and Prebiotics, Centennial, CO, United States; ^9^National Dairy Council, Rosemont, IL, United States

**Keywords:** fermented foods, probiotics, prebiotics, synbiotics, postbiotics

## Abstract

Fermented foods are often erroneously equated with probiotics. Although they might act as delivery vehicles for probiotics, or other ‘biotic’ substances, including prebiotics, synbiotics, and postbiotics, stringent criteria must be met for a fermented food to be considered a ‘biotic’. Those criteria include documented health benefit, sufficient product characterization (for probiotics to the strain level) and testing. Similar to other functional ingredients, the health benefits must go beyond that of the product’s nutritional components and food matrix. Therefore, the ‘fermented food’ and ‘probiotic’ terms may not be used interchangeably. This concept would apply to the other biotics as well. In this context, the capacity of fermented foods to deliver one, several, or all biotics defined so far will depend on the microbiological and chemical level of characterization, the reproducibility of the technological process used to produce the fermented foods, the evidence for health benefits conferred by the biotics, as well as the type and amount of testing carried out to show the probiotic, prebiotic, synbiotic, and postbiotic capacity of that fermented food.

## 1. Introduction

The International Scientific Association for Probiotics and Prebiotics (ISAPP) is a non-profit organization created in 2002 for the purpose of advancing the science of probiotics, prebiotics and related substances. In the period of 2014–2021, ISAPP proposed to the scientific, academic, health professional, industrial, and regulator communities a series of definitions, including probiotics (Hill et al., 2014), prebiotics (Gibson et al., 2017), synbiotics (Swanson et al., 2020), postbiotics (Salminen et al., 2021) (collectively referred to as the “biotics” or “the biotics family”) and fermented foods (Marco et al., 2021). The first four biotics definitions have a common theme in that a health benefit is expected following the administration of adequate amounts of a given biotic. However, a health benefit is not required of a fermented food. At the 2022 ISAPP meeting, a discussion group focused on *The Status of ‘Biotics’ in Fermented Foods* explored the variety of ways in which fermented foods might act as a delivery vehicle for various biotics. This perspective paper reports the outcomes of that discussion, including a brief review of fermented foods and health, the rising interest in fermented foods, the extent to which fermented foods are sources of biotics, and challenges to moving this field forward.

## 2. Fermented foods and health

Fermented foods have been part of the human diet from early civilization. Fermentation transforms foods, serving to preserve them from spoilage, enhance safety, improve nutritional value, and to provide unique, widely enjoyed organoleptic properties. These beneficial properties have led to the development of a various fermented products created from animal and vegetable sources. Increasing evidence of the association of fermented foods with health has further increased their popularity in recent years (Şanlier et al., 2019).

The evidence that fermented foods can confer health effects has been derived from, among other sources, randomized controlled trials involving defined interventions (ATLAS Collaboration et al., 2013; Kato-Kataoka et al., 2016; Lisko et al., 2017) or associative studies assessing dietary intake of fermented foods with often undefined microbial composition [for reviews, see (Marco et al., 2017; Dimidi et al., 2019)]. The most studied fermented foods are fermented milks, including yoghurt (Gómez-Gallego et al., 2018; Savaiano and Hutkins, 2021), which have been reported in multiple studies to have immunomodulatory effects, to promote lactose tolerance and gut transit, to help manage mild gut symptoms, to contribute to the prevention of osteoporosis, diabetes, and cardiovascular diseases (Hadjimbei et al., 2022), gastrointestinal infections, the reduction of serum cholesterol levels (Shiby and Mishra, 2013) and to impact the gut microbiota in a putatively beneficial manner (González et al., 2019). Despite health benefits being attributed to many fermented foods, in general, the evidence available does not allow us to discern which specific food component/s, or combination/s of components, are responsible for the observed health benefits.

The variety of food matrices, fermentation microbes, and technologies used, as well as the lack of knowledge on the composition and characteristics of some traditional fermented products, present a challenge in assigning a health benefit to a specific component or components of a fermented food. In addition to the macro- and micro-nutrients present, the beneficial effects of fermented foods are often attributed to the presence of different bioactives such as phenolic compounds, prebiotic-like substrates, and the microorganisms involved in the fermentation process. Some of these components may promote health by their ability to modulate the gut microbiota and/or to interact with the immune system of the host (Chakrabarti et al., 2014; De Marco et al., 2021; Rousseaux et al., 2023). Indeed, the increasing awareness of the role of intestinal microbiota in the etiopathology of different chronic diseases (Duvallet et al., 2017) makes the microbiota modulating potential of fermented foods a matter of increasing scientific interest. Some beneficial effects of fermented foods might be explained by such a modulating capacity (Kok and Hutkins, 2018).

A commonality among most fermented foods is the presence of microbes together with other known bioactives, including potentially prebiotic compounds, either already present in the food matrix or, in some cases, produced by the microorganisms during the fermentation process. In addition to the live bacteria responsible for the fermentation process, dead cells will also be present in fermented foods because of losses of viability during the starter/supplementary culture manufacture and/or a reduction in viability during product processing and storage. Such dead microbes could constitute postbiotic-like substances. For example, there is evidence that the administration of increasing amounts of different fermented foods can modulate the gut microbiota and lead to a decrease of biomarkers of inflammation (Wastyk et al., 2021).

## 3. The interest on fermented foods is increasing

Consumer and scientific interest in fermented foods continues to grow in Western countries, driven in large part by their association with digestive health and the promotion of the gut microbiome. Nearly one in four Americans cites digestive health as the most important aspect of their overall health (International Food Information Council, 2022) and online search inquires for the terms “gut health” and “microbiome” have increased by close to 70-fold and 10-fold, respectively, over the past 10 years. Fermented food and beverage sales reflect this increased consumer demand, with the global market projected to reach $989.2 billion by 2032 (Future Market Insights, 2022). Yoghurt, cheese, wine, beer and certain breads dominate the fermented food and beverage landscape, whereas interest in Kombucha, kimchi, tempeh and fermented sauces is growing quickly. Kimchi sales, for example, nearly doubled in 2020 alone, experiencing a 90% growth increase (Nielson-Stowell, 2020). The scientific community has increasingly turned its attention towards fermented foods, gut health, and the microbiome.

While fermentation has been used for thousands of years as a preservation (Taylor et al., 2020) method, its recent popularity surge is related to its potential to provide two of the most well studied components of gut health and microbiome support, probiotics, and prebiotics (Davani-Davari et al., 2019), as well as the lesser studied postbiotics. Probiotic fermented foods have been linked to various health benefits, from digestive health to immune support (Kim et al., 2019), and one in three Americans reported actively trying to consume probiotics in 2021 (International Food Information Council, 2022). Most consumers seek probiotics to support gut health, overall health and/or immune health (International Food Information Council, 2022). Two other biotics, prebiotics, and postbiotics, are beginning to draw attention for their health benefits as well, with 22% of Americans familiar with and actively trying to consume prebiotics followed by 13% for postbiotics (International Food Information Council, 2022). Despite the increased interest in fermented foods, confusion exists among consumers around their exact role in delivering probiotics, prebiotics, and postbiotics. For example, few fermented foods and beverages contain microbes that meet the criteria necessary to classify them as probiotics, yet a national consumer survey commissioned by Danone North America in 2022 reported that more than half of Americans (52%) believe that fermented foods do contain probiotics (personal communication). Another example of the confusion that exists is around prebiotics. Many prebiotic seekers look to yoghurt and kefir to provide these nutrients. However, few contain prebiotics (International Food Information Council, 2022). While the most studied prebiotics (i.e., inulin, fructooligosaccharides (FOS), and galactooligosaccharides (GOS)) are occasionally added to some fermented foods such as yogurt, other fermented foods may inherently contain some potential prebiotics. In fact, inulin-type fructans are found in varying concentrations within some raw vegetables (Muir et al., 2007; Kalala et al., 2018). For example, falsify, jerusalem artichokes, artichokes, onions, garlic, and leeks, they all contain inulin-type fructans. Thus, fermented versions of these vegetables would provide a source of prebiotics. However, cabbage, one of the most commonly fermented vegetables (i.e., kimchi, sauerkraut), does not contain detectable levels of prebiotic fructans, nor do cucumbers (pickles) (Muir et al., 2007). Ultimately, at present, few fermented foods contain accepted prebiotics and even fewer have been studied using randomized controlled trials to demonstrate the selective effects of the prebiotic substrate on the microbiota and the subsequent connections with health outcomes.

## 4. Are fermented foods a source of multiple biotics? Dairy as a case study

Can fermented foods be a source of probiotics, prebiotics, and postbiotics? The most studied category of fermented foods is fermented dairy products, with epidemiological studies demonstrating that the consumption of yoghurt is linked with reduced risk of metabolic disorders, cardiovascular disease, immune-mediated diseases or cognitive decline, among others (Guo et al., 2017; Salas-Salvadó et al., 2017; Panahi et al., 2018; Sivamaruthi et al., 2018). Yoghurt, which is produced by a symbiotic culture of *Streptococcus thermophilus* and *Lactobacillus delbrueckii* subsp. *bulgaricus*, should have a minimum of 10^7^ CFU/g until the expiration date. The European Food Safety Authority (EFSA) has approved a health claim for yoghurt containing live bacteria related to the improvement of lactose digestion in individuals with lactose maldigestion, illustrating the importance of the live microbes in yoghurt on a specific health outcome (Mukherjee et al., 2022). To bear the claim, the yoghurt should contain at least 10^8^ CFU/g of live starter microorganisms at the end of the shelf life.

**Figure 1:**
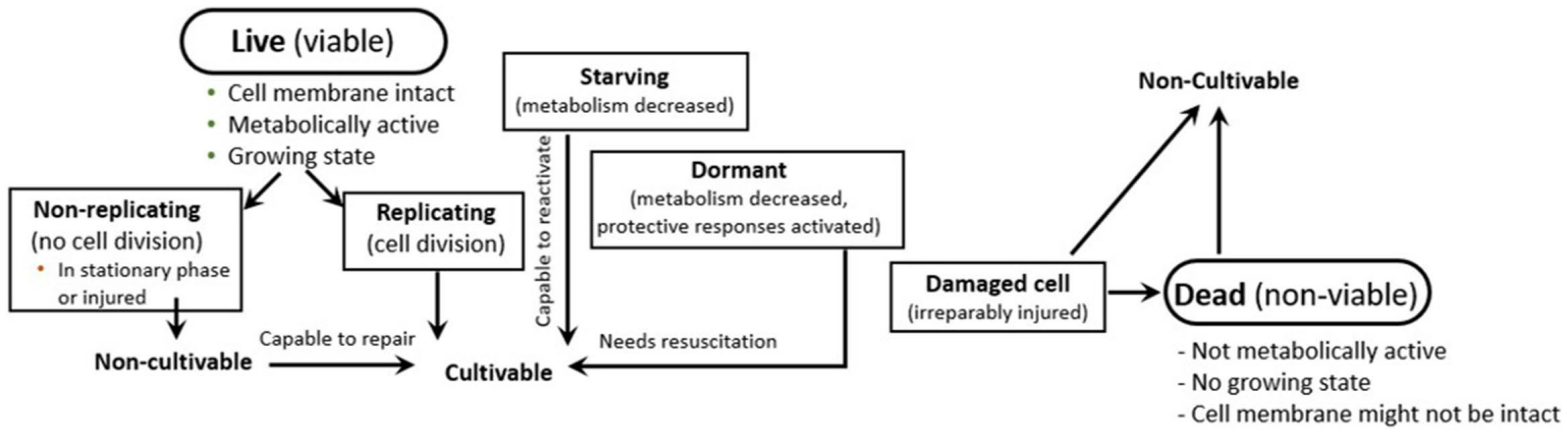
Different possible physiological states of a microorganism in a fermented food.

Yoghurt containing live bacteria may also deliver a proportion of non-viable microorganisms, but the actual number is challenging to calculate. It is well-known that bacterial cultures in foods have cells that are at different physiological states, ranging from live and active to dead bacteria (Figure 1), but the current methods used to determine the amount or physiological impact of bacteria in foods do not quantify these different physiological states (Davis, 2014). The application of culture-independent techniques might help to determine a more accurate picture of the physiological states of the microorganisms delivered by a fermented food (Porcellato et al., 2015; Fan et al., 2021). Importantly, it is not known if the microbe-derived health benefits associated with yoghurt consumption result from only the culturable (live) microbial population. Using yoghurt as an example, active β-galactosidase might still be present in the cytoplasm of viable but non-cultivable bacteria and free, active lactase may be also present in yoghurt, released by dead bacteria, thus all together also contributing to improve lactose digestion. Accordingly, yoghurt containing a certain population of viable but non-cultivable bacteria and/or dead bacteria, or a heat-inactivated yoghurt or fermented milks, could be considered a source of putative postbiotics with health benefits (Ouwehand and Salminen, 1998). This is just hypothetical unless proper studies are conducted to fit the definition of postbiotics. Finally, it has been described that some yoghurt commercial starters are capable of synthesizing exopolysaccharides (EPS) during milk fermentation, acting as natural biothickeners (Tiwari et al., 2021). Some of the EPS synthesized by LAB might act as prebiotic substrates for beneficial intestinal microbiota (Salazar et al., 2016). Therefore, both live- and heat-treated yoghurts could be a source of potential prebiotics, if they reach the minimum concentration needed to deliver a health benefit.

In summary, a typical “yoghurt” is an example of a fermented food potentially delivering multiple biotics, if minimum criteria are met for each biotic. The continuous ingestion of a fermented milk prepared with *Lactobacillus gasseri* CP2305, inactivated by heat treatment, showed efficacy in regulating the intestinal function in healthy individuals with a tendency toward constipation (Sawada et al., 2016), thus meeting the criteria to be considered as a postbiotic fermented milk. Following the ingredient approach, adding the well-known probiotic *Lacticaseibacillus rhamnosus* GG (Capurso, 2019) and the well-known prebiotic inulin (Hughes et al., 2021) to that product, a ‘multi-biotic’ fermented milk could be envisioned.

An important question to consider is whether it is useful to define different categories of “multi-biotics.” New terms and definitions need thoughtful discussion to determine, first, if they are needed, and second if they would be properly understood by stakeholders, including regulators, in a context of a growing list of “biotic” terms. However, it is beyond the scope of this perspective article to speculate on proposing new terms to name different possible combinations.

## 5. The microbiological complexity of some fermented foods may be a challenge for the delivery of biotics

While differences in the microbial consortia present across different fermented foods have an impact on the health effects and other attributes, such as flavor and texture, of those foods, important differences also exist within different versions of the same type of a particular fermented food. Milk kefir can be used as an example to demonstrate this point. Kefir is made when kefir grains [i.e., a consortia of bacteria and yeast contained in a polysaccharide (kefiran) matrix] are combined with milk and allowed to ferment (Bourrie et al., 2016). Then, the grains are removed to begin a new fermentation, while the milk is consumed or stored. It has been demonstrated that, even when the same kefir grains are used, the microbial composition of the resultant kefir can change daily (van de Wouw et al., 2021). Given that such heterogeneity is evident when a single grain is used, it is not surprising that even greater heterogeneity is apparent when different kefir grains are used (Marsh et al., 2013; Walsh et al., 2016). These differences can result in different volatile profiles (Walsh et al., 2016) and, even more importantly, different health benefits (Bourrie et al., 2018). For one set of 14 kefir grains, this has been demonstrated with respect to the capacity of different grains, or more accurately the microbes therein, to beneficially impact cholesterol, liver triglycerides, and weight gain in mice fed a high-fat diet (Bourrie et al., 2018). More specifically, it was established that only five of the 14 grains assessed were able to induce a significant drop in milk cholesterol and, when four of these were investigated further, only two brought about a significant reduction in plasma cholesterol, plasma non-HDL cholesterol and weight gain, and only one significantly reduced liver triglycerides, relative to controls fed non-fermented milk or a commercial kefir product (Bourrie et al., 2018). The importance of the associated microorganisms was further demonstrated when construction of a ‘pitched’ kefir, containing a defined combination of nine strains, was found to recreate these beneficial effects. However, versions of the defined community that lacked yeast or lactic acid bacteria were not effective (Bourrie et al., 2021). Some, but not all, of the health benefits were retained when cell-free or heat-treated fractions of the pitched kefir was used (Bourrie et al., 2022). The differing impacts on health effects due to differences in undefined kefir communities has also been highlighted in other studies, including, for example, investigations of the impact of kefir on the gut-brain axis (van de Wouw et al., 2020, 2021). These findings have not been confirmed in humans.

## 6. Future needs

For a fermented food to be labeled as able to deliver a ‘biotic’, the product must be sufficiently characterized and tested beyond the requirement for demonstrated health benefits. In this context, the term ‘probiotic’ should be reserved for live microorganims within fermented foods when there is a demonstrated health benefit conferred by the well-defined and characterized live microorganisms (Marco et al., 2021). Similar to other functional ingredients, the health benefits must go beyond that of the product’s nutritional components and food matrix. Therefore, the ‘fermented food’ and ‘probiotic’ terms may not be used interchangeably. This concept would apply to the other biotics as well.

To use the biotic term(s), there must be demonstration of health benefits in the target host and adequate characterization of the material(s) serving in that role. Biotic ingredients must be well-defined and characterized, proven to be safe and stable. For probiotics, this relates to the safety, identity, purity, and potency of the live microorganism in question (Hill et al., 2014; Swanson et al., 2020). Genome sequencing must be used to annotate and name the microorganism using current taxonomic nomenclature. This also allows for the assessment of genes relevant to safety (e.g., toxin production, antibiotic resistance). For prebiotics, characterization pertains to the structure and purity of the substrate in question. Purity level, which is known to vary greatly depending on source from 35 to 99% (Crittenden and Playne, 1996; Porras-Domínguez et al., 2019; Srivastava and Mishra, 2019), impacts performance and dosage required to demonstrate efficacy. Further, selective utilization by the host microbiota must be demonstrated concomitantly with the health benefit. For synbiotic preparations that contain a combination of probiotics and prebiotics, this same level of characterization and stability is expected. Postbiotics require molecular characterization of the progenitor microorganisms (similar to that of probiotics), a detailed description of the inactivation procedure and matrix, and a detailed compositional description of the final preparation (Salminen et al., 2021).

Based on these principles, sufficient characterization and testing would be required to label and/or market a fermented food or beverage with inclusion of a biotic term. While some manufacturers may be interested in using an individual term (e.g., probiotic fermented food), others may choose to combine more than one biotic substance similar to the synbiotic concept.

## 7. Conclusion

Fermented foods and biotics (probiotics, prebiotics, synbiotics, and postbiotics) are gaining attention as nutritional means to support health. Fermented foods is a broad category of foods that may contain live and defined strains of microorganisms (e.g., yoghurt), live and undefined consortia of microbes (e.g., kefir, kombucha, kimchi, and sauerkraut), non-viable defined (e.g., heat-treated yoghurt) or non-viable undefined microbes (e.g., pasteurized sauerkraut) or can be even devoid of significant amount of microbes (e.g., wine, beer). In this context, the capacity of fermented foods to deliver one, several, or all biotics defined so far will depend on the microbiological and chemical level of characterization, the reproducibility of the technological process used to produce the fermented foods, the evidence for health benefits conferred by the biotics, as well as the type and amount of testing carried out to show the probiotic, prebiotic, synbiotic, and postbiotic capacity of that fermented food.

## Data availability statement

The original contributions presented in the study are included in the article/supplementary material, further inquiries can be directed to the corresponding author.

## Author contributions

GV wrote the introduction and conclusions, compiled contributions, and drafted the paper. PC and HH covered the complexity of fermented foods section. MF covered the market aspects. MG and PR-M addressed the biotics perspective of fermented milk section. SS and KS covered the health benefits section. MS and CC discussed the outline of the paper and made the comprehensive revision and editing. All authors contributed to the article and approved the submitted version.

## Funding

ISAPP provided the venue and travel funding for some participants who met to discuss this topic, and paid for publication charges for this article.

## Conflict of interest

GV and SS serves in the board of directors of ISAPP and have been speakers in scientific meetings supported by the fermented food industry. PC has been funded by PrecisionBiotics Group, Friesland Campine, Danone, and PepsiCo. He has also received funding to travel to or present at meetings by H&H, the National Dairy Council U.S., PepsiCo and Yakult. MF is the employee of Health and Scientific Affairs, Danone North America and board member of the International Probiotics Association. KS serves in the board of directors of ISAPP. MS serves as the executive science officer/executive director for the International Scientific Association for Probiotics and Prebiotics (ISAPP), has been a paid consultant for Bayer, Pepsico, Smith Gambrell & Russell LLP, Bill and Melinda Gates Foundation, has been a paid speaker for Associated British Foods, European Federation of the Associations of Dietitians, Fairlife, Omnibiotic/Allergosan, Probi, Sanofi, Xpeer, has served on scientific advisory boards for Danone North America, Cargill, Winclove, has served in an unpaid (except for travel reimbursement) for the Advancement of Food and Nutrition Sciences and United States Pharmacopeia. CC is currently employed by the National Dairy Council. HH has received grant funding from Bio-Cat, Danone Research, General Mills, and Tate & Lyle. She has received speaking honorarium from Beneo, DSM, National Dairy Council, PepsiCo, and Tate & Lyle.

The remaining authors declare that the research was conducted in the absence of any commercial or financial relationships that could be construed as a potential conflict of interest.

## Publisher’s note

All claims expressed in this article are solely those of the authors and do not necessarily represent those of their affiliated organizations, or those of the publisher, the editors and the reviewers. Any product that may be evaluated in this article, or claim that may be made by its manufacturer, is not guaranteed or endorsed by the publisher.
